# Reproductive and Maternal Health in the Post-2015 Era: Cervical Cancer Must Be a Priority

**DOI:** 10.1371/journal.pmed.1001499

**Published:** 2013-08-13

**Authors:** Ruby Singhrao, Megan Huchko, Gavin Yamey

**Affiliations:** 1 Global Health Sciences, University of California San Francisco, San Francisco, California, United States of America; 2 Department of Obstetrics, Gynecology and Reproductive Sciences, University of California San Francisco, San Francisco, California, United States of America; 3 Evidence to Policy Initiative (E2Pi), University of California San Francisco, San Francisco, California, United States of America

## Abstract

Ruby Singhrao and colleagues propose four arguments for why cervical cancer screening and treatment should be prioritized.

*Please see later in the article for the Editors' Summary*

**Summary Points**

- Over the last two decades, there has been unprecedented global action on tackling high maternal mortality rates in low- and middle-income countries (LMICs) and on expanding access in these countries to reproductive health interventions, including family planning.
- The attention has paid enormous dividends: from 1990 to 2010, the annual number of maternal deaths worldwide fell from 546,000 to 287,000. While this positive trend is cause for celebration, we also believe that the global health community is failing women in a crucial way: it has neglected prevention, screening, and treatment for cervical cancer in LMICs.
- Such neglect is difficult to understand. Unlike some other cancers, including selected reproductive cancers, cervical cancer is detectable, highly preventable, and curable if detected early.
- There is an enormous international disparity in the incidence of, and survival from, cervical cancer, which are both also closely aligned with country income.
- The incidence of cervical cancer is 52.8 per 100,000 women in sub-Saharan Africa compared to 6.8 per 100,000 women in Western countries. Women are more likely to die in low-resource settings due to lack of infrastructure for screening.
- In this Essay, we propose four arguments for why cervical cancer screening and treatment should be included when it comes to operationalizing these two goals and thus to improving reproductive and maternal health outcomes. Each of the four arguments is illustrative of a larger framework that has equity and socioeconomic, gender, public health, and health services dimensions.

Over the last two decades, there has been unprecedented global action on tackling high maternal mortality rates in low- and middle-income countries (LMICs) and on expanding access in these countries to reproductive health interventions, including family planning [1]. Four major focusing events were particularly important in mobilizing awareness and funding. First, the International Conference on Population and Development, held in Cairo in 1994, provided the foundation for a comprehensive approach to women's health [2]. Second, in 2000, all 193 United Nations member states agreed to adopt Millennium Development Goal 5, which has both a maternal health and a reproductive health target. Target 5A is to reduce the maternal mortality ratio by three-quarters between 1990 and 2015, and Target 5B is to achieve “universal access to reproductive health” by 2015 [3]. Third, in 2010 the United Nations launched the Global Strategy for Women's and Children's Health, aimed at mobilizing US$40 billion to save the lives of 16 billion women and children over five years [4]. Finally, in July 2012, at the London Summit on Family Planning, donors pledged US$2.6 billion to scale up contraception [5], an important commitment given that about a third of all maternal deaths could be averted by scaling up comprehensive family planning services [4].

The attention has paid enormous dividends: from 1990 to 2010, the annual number of maternal deaths worldwide fell from 546,000 to 287,000 [6]. While this positive trend is cause for celebration, we also believe that the global health community is failing women in a crucial way: it has neglected prevention, screening, and treatment for cervical cancer in LMICs.

Such neglect is difficult to understand. Unlike some other cancers, including selected reproductive cancers, cervical cancer is detectable, highly preventable, and curable if detected early.

There is an enormous international disparity in the incidence of and rate of survival from cervical cancer, which are both also closely aligned with country income [7]. The incidence of cervical cancer is 52.8 per 100,000 women in sub-Saharan Africa, compared to 6.8 per 100,000 women in Western countries [8]. Women are more likely to die in low-resource settings because of lack of infrastructure for screening.

With the 2015 Millennium Development Goals deadline rapidly approaching, the international community is currently debating a “post-2015 development agenda,” which we believe should include taking global action on cervical cancer. The May 2013 report of the High-Level Panel of Eminent Persons on the Post-2015 Development Agenda recommends that there should be a single universal health goal (Goal 4) [9]. This goal would include reducing maternal mortality, ensuring “universal sexual and reproductive health and rights,” and reducing the burden of infectious diseases and “priority non-communicable diseases.” The panel also recommends a global goal on empowering girls and women and achieving gender equality (Goal 2).

In this Essay, we propose four arguments for why cervical cancer screening and treatment should be included when it comes to operationalizing these two goals and thus to improving reproductive and maternal health outcomes. Each of the four arguments is illustrative of a larger framework that has equity and socioeconomic, gender, public health, and health services dimensions (Figure 1). While we focus specifically on cervical cancer, we acknowledge that women in LMICs face a high burden of other cancers, such as breast and ovarian cancers.

**Figure 1:**
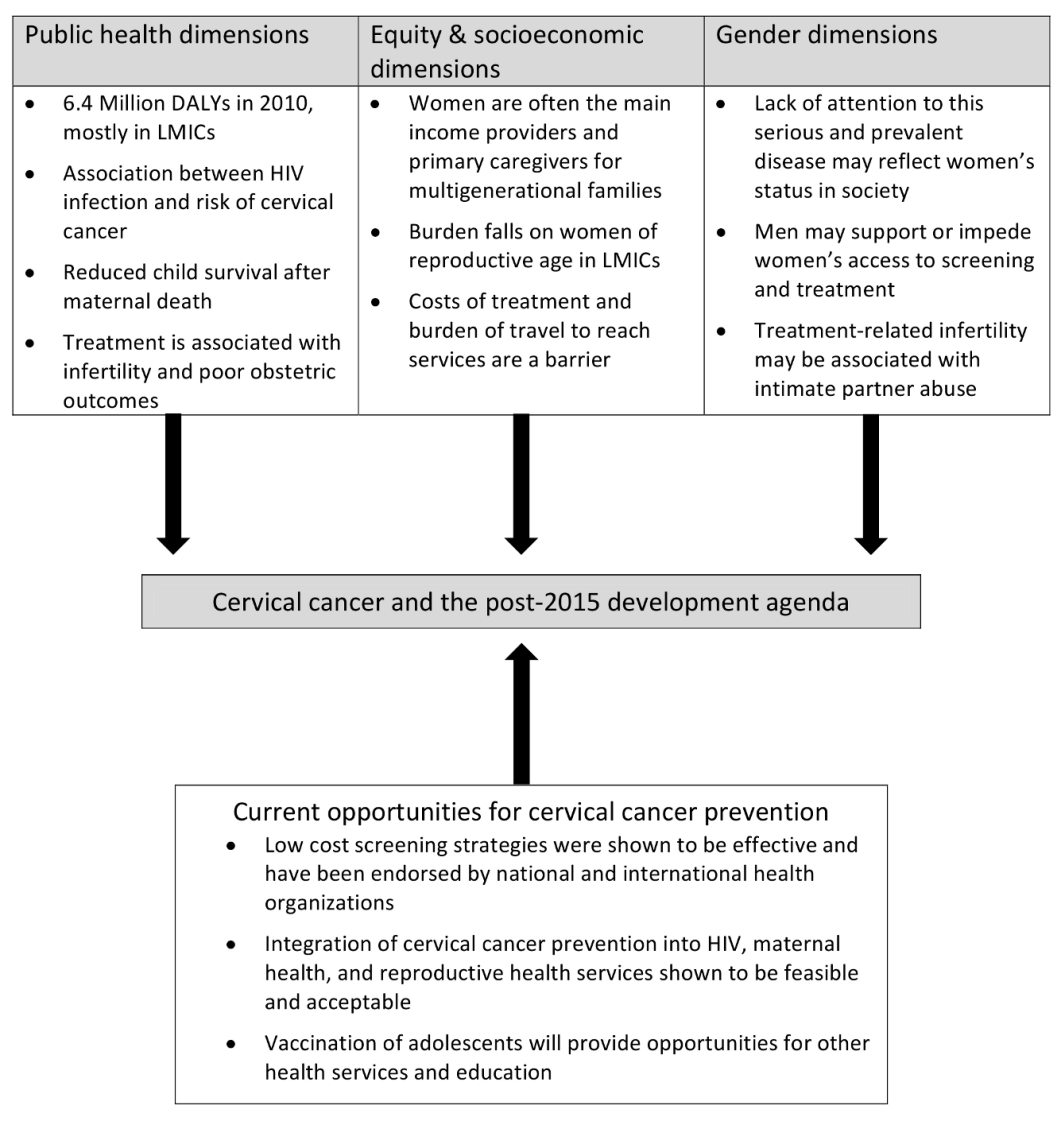
Cervical cancer and the post-2015 agenda: a multidimensional framework.

## The Burden Falls on Women of Reproductive Age

Our first argument is that the enormous global burden of cervical cancer falls mostly on women of reproductive age in LMICs. Each year, over half a million women globally are diagnosed with cervical cancer, and just over a quarter of a million die of the disease [8]. About nine in ten of these deaths occur in LMICs, where the mortality rate is 85% [10]. In many LMICs, women of reproductive age are the main income providers and primary caregivers for children and elderly relatives, and so these deaths have profound economic and social consequences.

Early maternal deaths from cervical cancer have intergenerational consequences for children, since children who lose their mothers are at higher risk of developmental delays and other poor health outcomes [11]–[13]. Two recent studies conducted in Bangladesh showed the detrimental impact of maternal death on child survival. The first study found that about six in ten children born shortly before the mother's death died in the first 90 months after her death [14]. The second found that children whose mothers die are three times less likely to survive to their tenth birthday compared to those with living mothers [15]. Maternal deaths also place an increased burden on extended family members, who often end up caring for the children.

In addition to the mortality consequences, the morbidity consequences of cervical cancer can profoundly affect a woman's quality of life. Women living with late stage cervical cancer may experience irregular bleeding, back pain, pelvic pain, fatigue, leg swelling, loss of appetite, and weight loss [16]. Early death due to cervical cancer and years affected by disease-related disability contributed to the loss of 6.4 million disability-adjusted life years (DALYs) globally in 2010, mostly in LMICs [17].

This burden of death and disability falls particularly heavily on sub-Saharan Africa, a situation related not only to the lack of preventive and treatment services, but also to the high HIV prevalence rate. HIV-infected women are at higher risk of developing cervical precancer and invasive cancer than uninfected women [18],[19].

The vast majority of women with cervical cancer in LMICs present to care when they have advanced disease. These women need the kind of treatment or palliative care only available at a major medical center [20]. For women in rural settings, the nearest tertiary care center can be located hundreds of miles away. In addition to the hospital fees, the costs of travel and lodging mean that poor women may not be able to afford treatment [21].

## Association with Reproductive Capacity

A second reason why cervical cancer should be central to Goal 4 of the post-2015 development agenda is the association between treatment for the disease and a woman's ability to conceive and deliver a healthy child. Treatments for cervical precancer and cancer are associated with increased risk of infertility and poor obstetric outcomes, including preterm delivery, low birth weight, and premature rupture of the membranes [22],[23]. Outpatient treatments can also lead to cervical stenosis in a small number of women, making pregnancy more difficult to achieve [24].

When available, the appropriate treatment for invasive cervical cancer includes a hysterectomy or radiation with chemotherapy [25], both of which render women infertile. Although fertility-sparing options have been explored in higher-income countries, they are limited to the small proportion of women with early invasive cancer, they require oncologic surgeons with specialized training and intensive follow-up, and they can result in poor obstetric outcomes [26],[27]. Such treatments are not widely available in most LMICs [27].

Infertility, a result of definitive treatment for cervical cancer, is associated with a high risk of familial and intimate partner abuse, depression, anxiety, and stigma [28]–[30]. The stigma of infertility may have an additional gendered dimension. In southern Ghana, for example, about two-thirds of women in one study reported feeling anxious and stigmatized by infertility, which included a fear of being expelled from the home by their husbands [31].

## Cancer Prevention Can Be Integrated into HIV, Maternal Health, or Reproductive Health Services

There is growing evidence of the feasibility of integrating cervical cancer prevention into HIV, maternal health, or reproductive health services using low-cost screening strategies coupled with treatment for precancerous lesions [32]. Conventional screening methods, using Pap smears and biopsies, require infrastructure and clinical expertise and are hard to scale up in LMICs. But simpler, cheaper screening techniques, such as visual inspection with acetic acid (VIA) and human papillomavirus (HPV) DNA testing, hold great promise and are undergoing widespread evaluation [33].

The World Health Organization global action plan on noncommunicable diseases describes screening with VIA as a “best buy,” meaning that it is both highly cost-effective (i.e., it costs less than the per capita gross domestic product to avert one DALY) and feasible to implement in settings with constrained health systems [34]. There are promising results from large trials suggesting that VIA can reduce cervical cancer incidence by 25%–30% [35],[36]. Although screening with HPV DNA testing is more expensive than with VIA, a study by Goldie and colleagues in five LMICs found that HPV DNA screening is very cost-effective, and a single test at age 35 years reduces lifetime cancer risk by 25%–36% [37]. Integrating screening into primary care services for women should increase the likelihood that precancer is detected, as is seen in high-income countries, where effective screening averts progression to cervical cancer.

Integrating care for HIV, sexual health, reproductive health, and maternal health has been shown to improve uptake of services, reduce HIV-related stigma, and improve the quality of care received by women [38],[39]. A recent study in western Kenya showed that it was feasible to integrate cervical cancer screening into HIV outpatient clinics [40].

Furthermore, integrating cervical cancer prevention services into primary care facilities provides an opportunity to include and educate male partners, which may be particularly important in regions where men have control over health care decisions [41],[42].

## HPV Vaccination Can Protect Girls from a Fatal Disease

Finally, almost all women with cervical cancer are infected with HPV. The World Health Organization has approved two HPV vaccines that could dramatically reduce cervical cancer deaths in LMICs if vaccination coverage can be scaled up [43]. A recent multinational trial showed that the vaccine can reduce precancerous lesions by up to 90% [44].

Ensuring that adolescent girls have the opportunity to receive a vaccine that protects them from death, infertility, and other morbidity related to cervical cancer, should be a key global health priority. Vaccination of adolescent girls also provides an opportunity to provide them with other reproductive health services and health education (including education on family planning and menstrual hygiene).

## Next Steps

Including cervical cancer in the post-2015 agenda would give the disease the policy priority that it deserves, and could help to attract greater domestic and international donor funding. Lowering the burden of cervical cancer in LMICs will not happen by chance—it requires national and international leadership, attention, and resource mobilization to roll out primary disease prevention through HPV vaccination programs, and secondary prevention through low-cost screening strategies. Rwanda is a good example of how prioritizing cervical cancer at the national level, mobilizing key actors (public, private, and international), and working across multiple sectors (including education) are effective in rolling out HPV vaccination [43]. The country's national campaign to vaccinate school girls achieved over 90% coverage rates.

An important step forward is that primary prevention through scaled up HPV vaccination will be rolled out in eight LMICs in 2013 (Ghana, Kenya, Madagascar, Malawi, Niger, Sierra Leone, Tanzania, and Lao People's Democratic Republic) through the support of the GAVI Alliance [45]. Julio Frenk, former Minister of Health of Mexico, and current dean of the Harvard School of Public Health, has argued that the GAVI Alliance's decision to include HPV vaccine in its financing portfolio is “a visionary investment that will improve the health of girls and women, equity, and development.”

The GAVI Alliance announced on May 9, 2013, that the world's poorest countries will be able to obtain, with its support, HPV vaccine for as low as US$4.50 per dose through the United Nations Children's Fund Supply Division [45]. While this low price is clearly good news, modeling by Goldie and colleagues in 72 countries eligible for this GAVI Alliance–supported program suggested that the intervention would become very cost-effective only at a price of about US$2 per dose [46]. Eventually, we look forward to an era in which all girls *and* boys are offered the public health benefits of HPV vaccination.

The health of women and girls must continue to be prioritized in the post-2015 development agenda. Assuming that the maternal mortality rate continues to fall, we are likely to see a continuing rise in chronic diseases in women in LMICs, including cancers. Thus, the post-2015 agenda needs to include a concerted global plan to curb the “cancer crisis” of developing countries. For cervical cancer, we fortunately now have a wide range of feasible, affordable, and effective prevention options, which make dramatic global reductions in cervical cancer incidence a realistic goal in our lifetime.

## Abbreviations

DALY: disability-adjusted life year
HPV: human papillomavirus
LMICs: low- and middle-income countries
VIA: visual inspection with acetic acid
